# Recurrent *PIK3CA H1047R*-Mutated Congenital Infiltrative Facial Lipomatosis: A Case Report and Review of Literature

**DOI:** 10.3390/cimb45020110

**Published:** 2023-02-17

**Authors:** Kei Shing Oh, Hisham F. Bahmad, Kalin Veselinov Stoyanov, Ibrahim H. Amjad, Carole Brathwaite

**Affiliations:** 1 The Arkadi M. Rywlin M.D., Department of Pathology and Laboratory Medicine, Mount Sinai Medical Center, Miami Beach, FL 33140, USA; 2 Department of Translational Medicine, Herbert Wertheim College of Medicine, Florida International University, Miami, FL 33199, USA; 3 Amjad Plastics, Miami, FL 33144, USA; 4 Department of Pathology and Laboratory Medicine, Nicklaus Children’s Hospital, Miami Children’s Health System, Miami, FL 33155, USA

**Keywords:** lipomatosis, *PIK3CA*, surgery, face

## Abstract

Congenital infiltrating lipomatosis of the face (CILF) is a rare, congenital, nonhereditary facial overgrowth due to post-zygomatic activating mutations in *PIK3CA* gene. It is unilateral and involves hypertrophy of both the soft and hard tissue structures on the affected side of the face. This commonly results in early eruption of the teeth, hypertrophy of the facial bones, macroglossia, and proliferation of the parotid gland. Less than 80 cases of CILF have been reported in the literature so far. Treatment modalities include liposuction and surgical excision. However, since the hallmark of CILF is mutation in the *PIK3CA* gene, PI3K inhibitors may play a therapeutic role in CILF. We report a case of an 8-year-old boy with recurrent CILF of the scalp and nose, with *PIK3CA H1047R* mutation. We discuss the differential diagnoses, clinical outcomes, and management of this rare entity.

## 1. Introduction

Congenital infiltrating lipomatosis of the face (CILF) is a rare, congenital, nonhereditary, unilateral facial overgrowth [1]. Although it is not proven to be neoplastic through monoclonality, it occurs due to post-zygomatic activating mutations in the *PIK3CA* gene, constituting a part of *PIK3CA*-related overgrowth syndrome. This causes mature lipocytes to invade into the adjacent tissues in the facial region [2,3]. The *FGFR3* gene has also been shown to instigate a role in the etiopathogenesis of CILF [4]. This lesion is characterized as a benign lesion with an indolent growth pattern, yet it is known to be highly recurrent, requiring multiple debulking surgeries. It mainly affects the pediatric population.

CILF was first described by Slavin et al. in 1983 [5]. Histologically, it is characterized by proliferation of nonencapsulated, mature lipocytes that locally infiltrate facial muscles, soft tissues, and bones causing ipsilateral hyperplasia of the facial skeleton, asymmetry of the mandible, and dental abnormalities [1,5]. Children with CILF are asymptomatic and have normal psychomotor development; aesthetic considerations are the main concern [1]. Here, we report a case of an 8-year-old boy with recurrent CILF of the scalp and nose, with *PIK3CA H1047R* mutation.

## 2. Case Presentation

**Figure 1:**
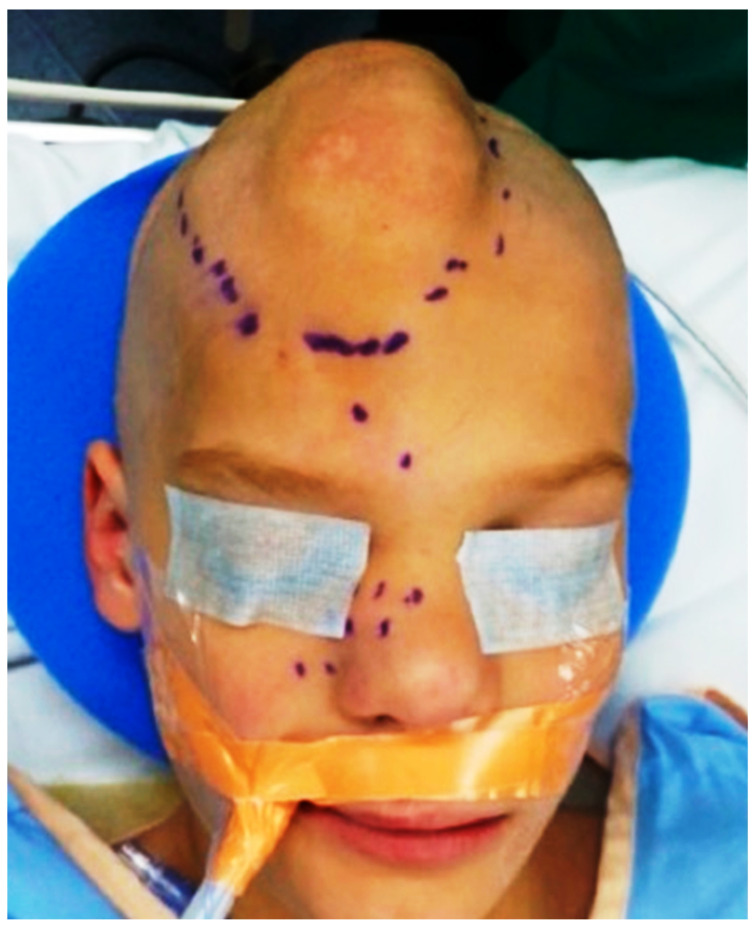
Ill-defined soft tissue mass in the patient’s forehead showing local extension to the scalp and inferiorly to the nose bridge and right lateral aspect of the nose.

An 8-year-old boy was brought into our institution with facial asymmetry progressively evolving since birth. He had a similar history of a subcutaneous scalp mass at birth and another mass at the right upper eyelid, glabella and scalp at the age of 2, which was removed surgically without any complication. Now, the patient presented with a recurrent mass involving the scalp and nose. There was no significant family history or any siblings with similar presentation. Physical examination showed an ill-defined soft tissue mass in the forehead extending to the scalp and inferiorly to the nose bridge and right lateral aspect of the nose (Figure 1). The patient had no known allergies and did not have any fever, nasal congestion, cough, wheezing, or shortness of breath. Physical examination did not show any other significant findings and his vital signs were within normal limits.

**Figure 2:**
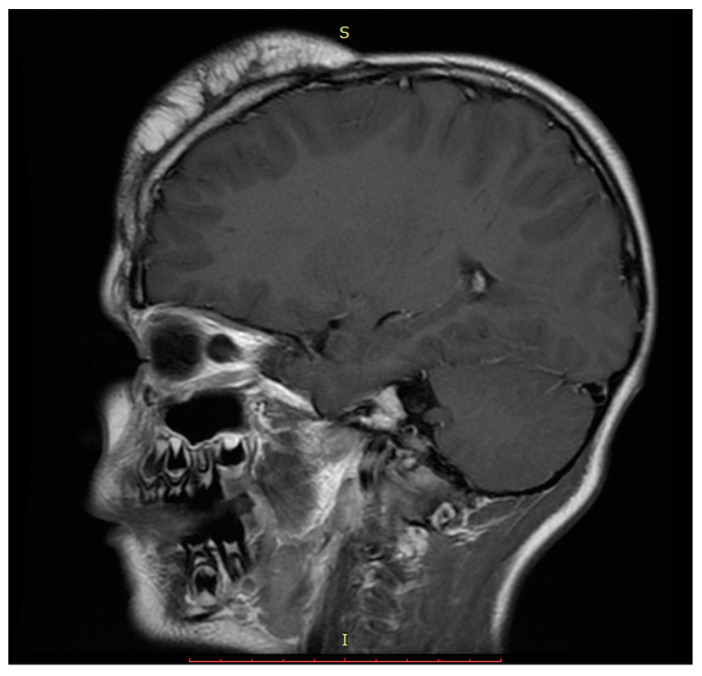
Sagittal T1 magnetic resonance image showing heterogenous lobulated fat in the high frontal scalp.

Head and face computed tomography scan and magnetic resonance imaging were performed and showed an enlarging soft tissue lesion measuring 9.7 × 7.4 × 3.1 cm in the forehead towards the right side extending to the parietal scalp. There was no involvement of the underlying skull bone, brain, or ventricle. The lesion was characterized primarily by fat signal with areas of soft tissue signal in the periphery, traversing the fatty component (Figure 2). Spiral computed tomography through the entire head and face were done with and without intravenous contrast. An enlarging soft tissue lesion is again noted in the forehead towards the right side extending towards the right parietal scalp. The lesion is characterized by primarily fat density component. Irregularity of the underlying bone is seen, however, no frank destruction of the underlying calvaria is seen. The differential diagnosis based on radiology findings includes growing lipoma, liposarcoma, or facial lipomatosis. 

Surgical resection of the mass via a bicoronal incision on the scalp was performed. Intraoperatively, dissection was carried down to the periosteum underneath the area of the level of the galea and the mass was noted. Careful and slow dissection was done to ensure complete removal of the mass and to preserve the intactness of the overlying skin and subcutaneous tissue. Due to the infiltrative nature of the mass which showed extensive local infiltration into the muscles, skin and galea, it was removed via a piecemeal approach. The patient tolerated the procedure well and was sent to recovery in stable condition.

**Figure 3:**
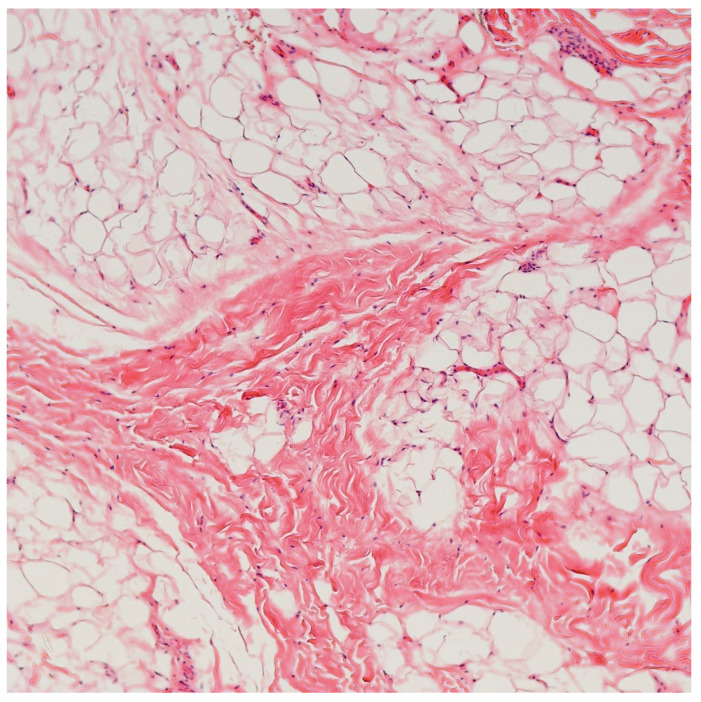
Microscopic images showing that the mass predominantly consisted of mature adipose tissue with thin and thick, irregular fibrous septae (Hematoxylin & eosin, 100×).

Macroscopically, multiple irregular fragments of focally hemorrhagic yellow to brown soft tissue with yellow-pink lobulated cut surface and focal white-tan fibrous elements. Histopathological examination showed that the mass consisted predominantly of mature adipose tissue with thin and thick, irregular fibrous septae (Figure 3). No atypia or increase in mitotic activity is noted. Based on the histological findings, the specimen was sent out to FoundationOne Heme, a comprehensive genomic profiling testing designed to identify genomic alterations in various cancer-related genes in hematologic and soft tissue malignancies. This test assay utilized DNA sequencing to examine 406 genes and the selected introns of 31 genes involved in rearrangements, as well as RNA sequencing of 265 genes. The entire coding sequence for the detection of base substitutions, insertion or deletions, and copy number alterations were studied. The mass revealed a mutation in the *PIK3CA H1047R* gene (Transcript ID: NM_006218, Coding sequence effect: 3140A>G), supporting the diagnosis of congenital infiltrative lipomatosis of the face. The tumor was microsatellite stable and there was no increase in tumor mutational burden (TMB) (0 mutation/Mb).

## 3. Discussion

Congenital infiltrating lipomatosis of the face (CILF) is a rare, congenital, nonhereditary facial overgrowth first described in 1983 by Slavin et al. [5]. There are less than 80 reported cases in the literature [6]. With the discovery of postzygotic mosaic activating mutations in the *PIK3CA* gene in CILF, it is now considered part of *PIK3CA*-related overgrowth syndrome [2]. Nevertheless, CILF with a *PIK3CA* gene mutation is rare. A review of the literature of select reported cases thus far has identified frequent *PIK3CA* hotspot mutations including *H1047R*, which is the most frequent drive mutation hotspot, as in our case, and other hotspots such as *H1047L*, *E453K*, *E452K*, and *E542K* (Table 1).

Additionally, a literature review was performed using PubMed (National Institutes of Health, National Library of Medicine), EMBASE (Elsevier), and CIHNAL (EBSCO) using multiple variants of common search keywords (MeSH terms) such as “CILF,” “FIL,” “*PIK3CA* mutations,” “Congenital Infiltrating Facial Lipomatosis,” and “Facial Infiltrating Lipomatosis.” The subsequent literature review showed that prior to 2013, no reported cases of CILF with a confirmed *PIK3CA* mutation are found. This is likely due to the more recent discovery of *PIK3CA* as a driver mutation for pathogenesis of CILF, and the natural delay in translating this discovery towards actual clinical testing performed to confirm the diagnosis in studied patients. Thus, previously published cases not including confirmed *PIK3CA* mutations were excluded from this review. The review of literature produced 16 case reports in a total of 7 publications. While no language restrictions were used, the utilized articles were all published in English.

Table 1 displays 15 selected cases of CILF with confirmed somatic *PIK3CA* mutations. This collated search revealed the importance of increasing molecular testing in patients with suspected CILF, as the vast majority of recently published literature on CILF unfortunately does not include molecular testing as a confirmatory approach. While the knowledge of this mutation existing in our patients does not necessarily change the surgical approach and management of CILF, it does reveal that potential various molecular therapeutics may be used if they happen to interrupt the aberrant *PIK3CA* pathway involved in the pathogenesis of CILF. These new potential therapeutics will be briefly described later in our discussion. We hope and anticipate that further research utilizes molecular screening tools such as comprehensive genomic profiling or techniques such as the ones listed on Table 1 to aid our understanding of this illness.

Previous research has notably shown that CILF does not show any sex or side predilection. In our findings shown on Table 1: 8 out of 15 (8/15) reported cases list the gender of the patient, and 6 out of 8 (6/8) patients were female. In some of the listed cases, such as the one by Couto et al. [7], the age was included as the “age [in years] of operation.” Other listed cases such as Kalantary et al. [8] described a much more detailed timeline in which the patient was followed, where longitudinal care and multiple treatments were provided. Seven cases reported the side of the lesion, of which, 4 cases were found on the left face, and 3 were found on the right face. Six cases have had surgery, while surgery was not explicitly listed for the remainder of the cases. Of the 3 listed cases where recurrence is described, CILF did not recur in 2 cases, and did recur in only 1 case. Most of the listed cases reported the method of molecular testing performed as either Polymerase Chain Reaction (PCR) or Double Droplet Polymerase Chain Reaction (ddPCR). Three cases reported *PIK3CA* mutations without explicitly reporting the molecular testing methodologies performed. However, of the publications that reported *PIK3CA* mutations as the cause of the CILF, 6 cases performed PCR testing to identify the mutations, and the other 6 cases performed ddPCR testing. In terms of the types of mutation, all except one case reported specific mutation detected. Missense substitution *p.H1047R* appears to be the most frequently found at 7 out of 15 cases (7/15)), followed by the second most frequent mutation reported being the missense substitution mutation of *p.E542K*, which was found in 4 of the 15 cases (4/15). The least frequent mutation reported in this review was *p.H1047L*, which was found in 3 out of 15 cases (3/15).

**Table 1:** Review of select congenital infiltrating facial lipomatosis cases with confirmed *PIK3CA* mutations.

Year [Ref]	Number of Cases	Gender	Age	Site	Surgery	Recurrence	Molecular Test	Mutation Detected
2013 [2]	6	N/A	N/A	N/A	N/A	N/A	PCR	*p.H1047R*
		N/A	N/A	N/A	N/A	N/A	PCR	*p.H1047L*
		N/A	N/A	N/A	N/A	N/A	PCR	*p.E452K*
		N/A	N/A	N/A	N/A	N/A	PCR	*p.E542K*
		N/A	N/A	N/A	N/A	N/A	PCR	*p.E453K*
		N/A	N/A	N/A	N/A	N/A	PCR	*p.H1047R*
2015 [9]	2	Female	4 years	Left face	N/A	N/A	ddPCR	*p.H1047R*
		N/A	N/A	N/A	N/A	N/A	ddPCR	*p.H1047L*
2017 [8]	1	Male	4–18 years	Right face	Yes	No	N/A	*N/A*
2017 [7]	3	Male	3 years	Right face	Yes	N/A	ddPCR	*p.H1047R*
		Female	8 years	Left face	Yes	N/A	ddPCR	*p.H1047R*
		Female	15 years	Left face	Yes	N/A	ddPCR	*p.H1047L*
2018 [10]	1	Female	8 years	N/A	N/A	N/A	ddPCR	*p.E453K*
2018 [11]	1	Female	5 years	Left face	Yes	Yes	N/A	*p.H1047R*
2020 [12]	1	Female	5 years	Right face	Yes	No	N/A	*p.H1047R*

Abbreviations: ddPCR: Droplet Digital Polymerase Chain Reaction; N/A: Not applicable; NGS: Next Generation Sequencing; PCR: Polymerase Chain Reaction.

Clinically, CILF presents as unilateral, progressive, painless enlargement of the soft and bony facial tissue structures and occurs at birth or in early childhood (under the age of one year). Notably, CILF does not show any sex or side predilection. Due to its extensive and infiltrative growth pattern, it may be associated with orodental issues such as macrodontism, early eruption of deciduous and permanent teeth, and facial deformities, which entail a significant social and psychological impact [6]. Histologically, CILF is characterized by an unencapsulated mass composed of mature adipose tissue without atypia, mitosis, or lipoblasts, with intervening fibrous tissue septae that infiltrates adjacent muscles and soft tissues, with associated hypertrophy of the underlying bone, making complete excision extremely difficult [13].

Postzygomatic activating mutations in the *PIK3CA* gene are identified in CILF as well as other benign overgrowth syndromes, which are collectively termed *PIK3CA*-related overgrowth syndrome [2]. *PIK3CA* is a gene that encodes p110α, a catalytic PI3K subunit that has a physiological role in growth and development and frequently displays pathological hyperactivation in cancers [14]. With the use of high-throughput gene sequencing, genetic hyperactivation of PI3K/AKT signaling has now become well recognized as one of the common “driver” mechanisms in many solid cancers, including breast, endometrial, bladder, colorectal carcinoma, and head and neck squamous cell carcinoma [15,16,17]. However, benign overgrowth syndromes such as CILF occur because of postzygomatic activating mutations in *PIK3CA* with asymmetric overgrowth, exemplifying a mosaic genetic activation of p110α. Furthermore, *PIK3CA* hotspot mutations were also identified in benign skin lesions such as epidermal nevi and seborrheic keratosis [18], representing another example of genetic mosaicism. The cellular substrate underlying the aberrant tissue accumulation and the infiltrative nature of CILF remains unknown. However, in an in vivo study on mice, it was demonstrated that a population of neural crest-derived fibro/adipogenic progenitor cells differentiate into mature adipose tissue and infiltrate craniofacial tissue in response to tissue injury, in the presence of systemic or local pro-adipogenic signals [19]. This study reported that tissue injury, along with an obesity inducing diet might contribute to the proliferation and accumulation of craniofacial connective tissue, a phenomenon seen in CILF [19].

The diagnosis of CILF is mainly clinical. Imaging studies such as ultrasound, computed tomography scan, and magnetic resonance imaging may help to detect the presence of abnormal vascularity or any involvement of underlying bone and soft tissue structures. Histopathological examination of the biopsied or excised tissue helps to rule out other possible differential diagnoses in the facial region such as lipoma, lipoblastoma or liposarcoma. Lipoma is a common benign neoplasm of adipocytic differentiation and usually presents in adulthood. Lipoma is usually well encapsulated and follows a relatively indolent course, whereas CILF is non-encapsulated with ill-defined borders and tends to infiltrate extensively into surrounding tissues. Lipoblastoma, another benign entity which is commonly seen in young children, shows similar histopathology and is characterized by sheets of mature adipocytes, separated by delicate fibrovascular septae. However, lipoblastoma typically involves the trunk and extremities and rarely arises in the head and neck region. Furthermore, the diagnosis of lipoblastoma can be supported with *PLAG1* rearrangements or copy number gain, which is absent in our case. Lastly, the absence of lipoblast, atypical adipocytes, and mitosis can help to rule out a diagnosis of liposarcoma, which more commonly affects older population, and shows a predilection to involve the trunk and extremities.

CILF is an indolent benign tissue overgrowth with a high risk of recurrence, requiring multiple debulking surgeries. Of note, although CILF is associated with *PIK3CA* mutations, associations with malignancies are rare. The only reported malignancy in *PIK3CA*-related overgrowth syndrome is Wilms tumor (nephroblastoma) which is identified in four out of 200 patients with *PIK3CA*-related overgrowth syndrome [20]. Furthermore, although activating *PIK3CA* driver mutations can confer a selective growth advantage during cancer development, the mutation alone is not sufficient for cancer initiation or maintenance [21] and requires cooperating genetic lesions to induce cancer [22,23], an important explanation for the lack of associated cancer in patients with *PIK3CA*-related overgrowth syndrome and, likely, CILF.

In a review of literature by Li et al., incorporating all cases of CILF reported until 2017, 59 patients (ages ranging from 0 to 53 years old, mean age = 12 years old) were found [3]. All patients presented with facial asymmetry and facial soft tissue mass, and most of them had associated ipsilateral hypertrophy of the underlying bones [3]. In this study sample, the recurrence rate was 79%, with an average of 1.95 operations performed for each recurrent case. 

The diagnosis of this pathological entity is based on the clinical history, clinicopathological manifestations, imaging features from computed tomography of the head region, and genetic profiling of the excised tissue. Although there are not sufficient data about an increased risk of *PIK3CA*-associated adult cancers among *PIK3CA*-related overgrowth syndrome patients, cooperating mutations might still contribute to cancer development in this population of patients [24].

Treatment modalities currently available for CILF include mainly liposuction and surgical excision [3]. Although considered benign, CILF has a high recurrence rate post-excision (up to 62.5%) [25], requiring multiple surgeries for cosmetic reasons [26,27]. Additionally, new therapeutic approaches combining surgical and medical interventions, such as targeted chemotherapy with imatinib and celecoxib, have shown promising results with improvements in facial symmetry and without disease progression at 18 months follow-up [13]. Moreover, the involvement of *PIK3CA* gene mutation in CILF renders inhibitors of PI3K such as Alpelisib, which are currently under study in clinical trials for many cancers including lymphoma, a potential therapeutic option for CILF patients [2].

## 4. Conclusions

In conclusion, we report a case of recurrent CILF in an 8-year-old boy with *PIK3CA* gene mutation. This case highlights the need for heightened awareness of this rare disease among pathologists as well as the need to perform molecular studies on “seemingly benign”-appearing mature adipose tissues from the face and scalp of a child. 

## Data Availability

Not applicable.
